# Supplementary figures and images for: BiSulfite Bolt: A bisulfite sequencing analysis platform
Source: Gigascience. 2021 May 8;10(5):giab033. doi: 10.1093/gigascience/giab033 (PMC8106542; doi:10.1093/gigascience/giab033)

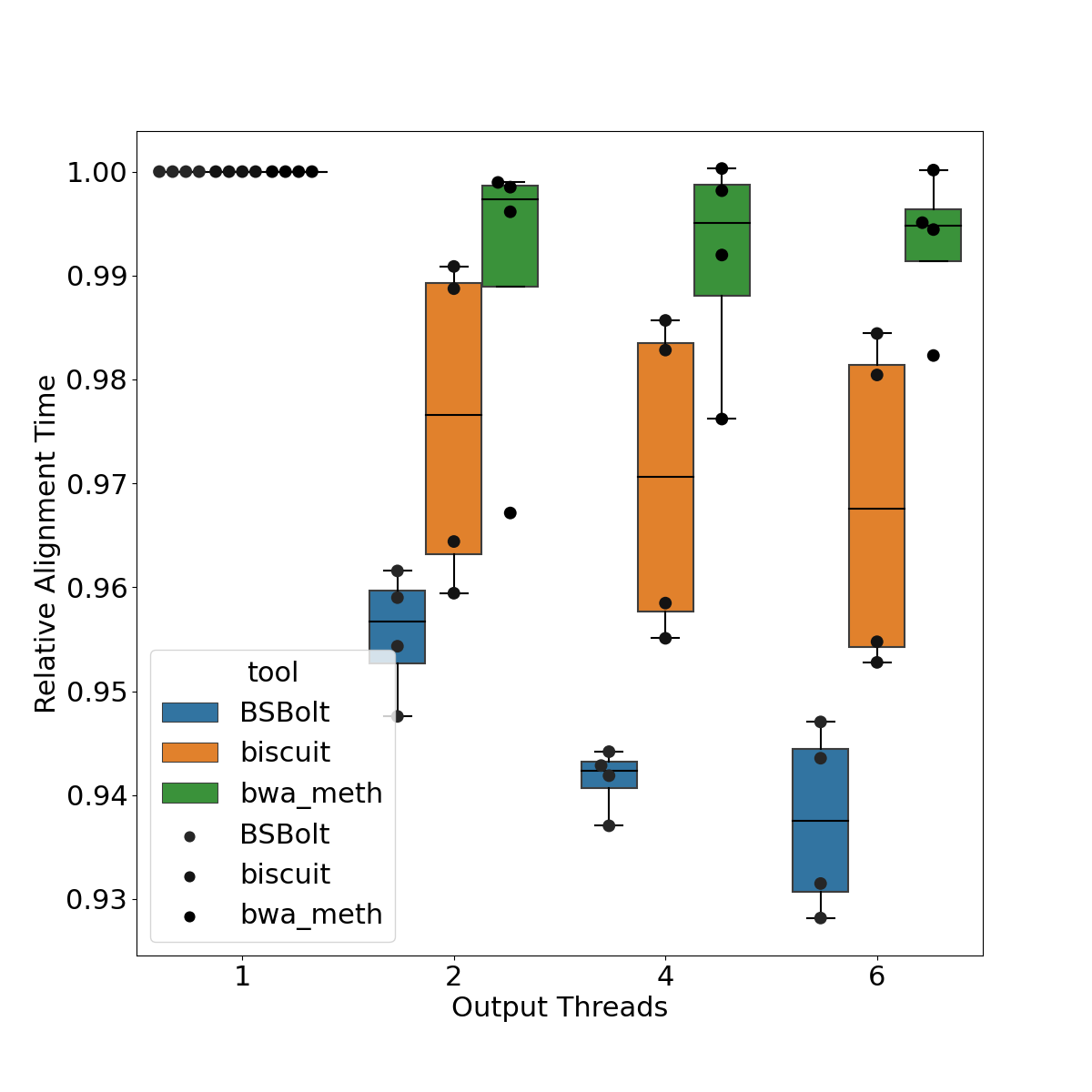

Supplement: giab033_Supplemental_Files [file giab033_supplemental_files.zip › SupplementalFigure1.png]

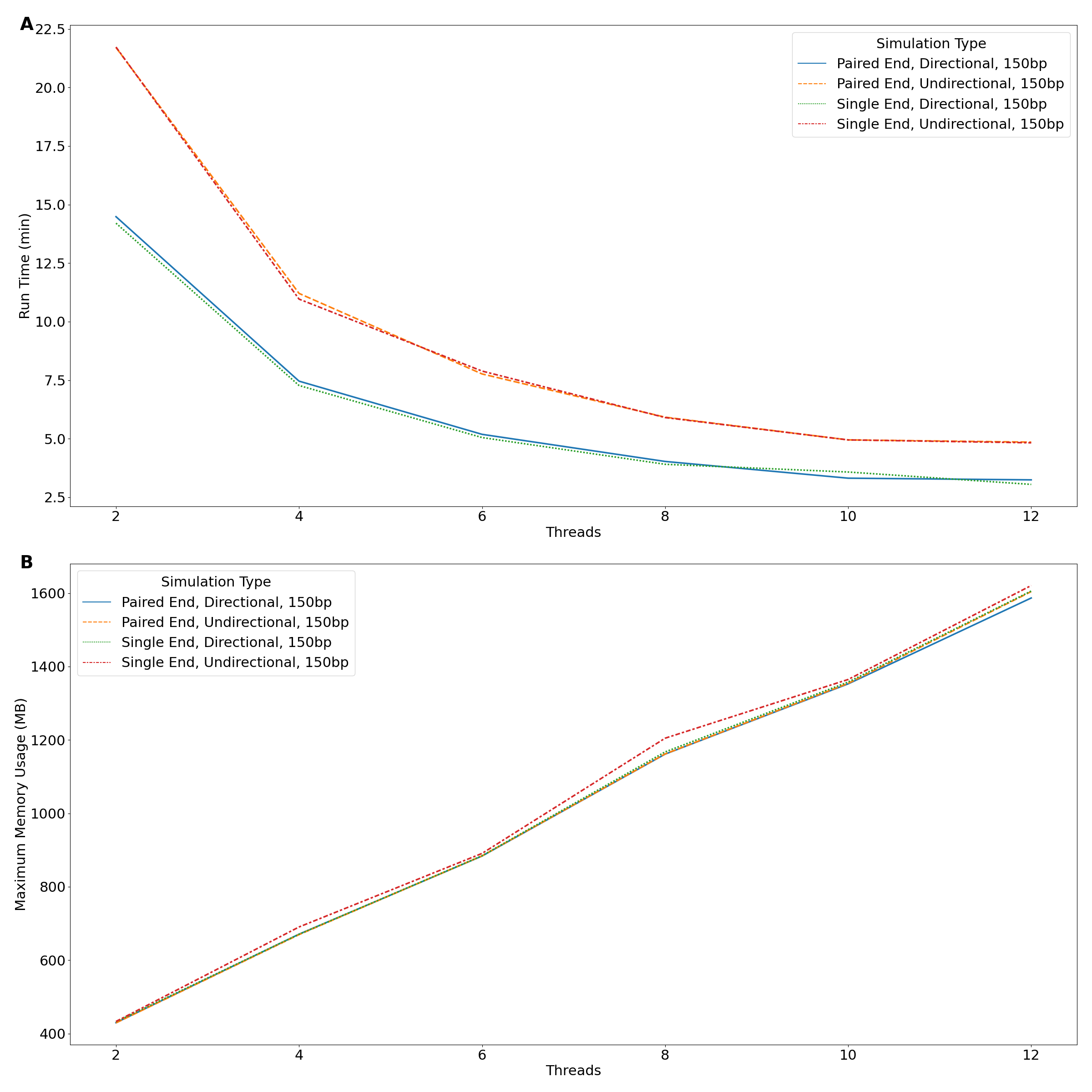

Supplement: giab033_Supplemental_Files [file giab033_supplemental_files.zip › SupplementalFigure2.png]
