# Supplementary material for: BiSulfite Bolt: A bisulfite sequencing analysis platform
Source: Gigascience. 2021 May 8;10(5):giab033. doi: 10.1093/gigascience/giab033 (PMC8106542; doi:10.1093/gigascience/giab033)
Supplement: giab033_Response_to_Reviewer_Comments_Original_Submission [file giab033_response_to_reviewer_comments_original_submission.pdf]

Author's Response To Reviewer Comments

Close

Dear Editor Zhou,

We wish to thank the reviewers for their useful comments. We have revised the manuscript to address the comments. Please find our point-by-point responses below, marked in blue.

Reviewer #1:

The authors introduce BSBolt as a complete pipeline for processing bisulfite sequence data.The main stated contribution of this tool over the authors' previous work of BSSeeker and BSSeeker2 is the direct modification of BWA-mem to align BS-Seq reads directly which results in increased accuracy. This seems like a nice improvement over existing methods. Several clarifications/changes in the paper would be helpful.

1. Given that the authors have previously written BSSeeker and recently written BSSeeker2, more concise motivation of what short-coming BSBolt addresses in specifically those two tools would be helpful.

Response:
We expanded on the motivation to develop BSBolt in the manuscript text below. In summary, we had several goals with BSBolt. We sought to improve performance, eliminate the alignment constraints enforced by BSSeeker / BSSeeker2, preserve the read level methylation calling and read bisulfite conversion checks, and finally we wanted to take a minimal performance hit for alignment of undirectional libraries.
The three base alignment strategy as implemented by BSSeeker2 and Bismark has several limitations. Both tools carry out multiple intermediary alignments to separate alignment indices representing different reference conversion patterns and then integrate intermediate alignments together into a consensus alignment file. Reads with multiple alignments within an intermediate alignment file or across multiple intermediate alignment files are discarded; only reads that align uniquely within a single intermediate alignment are reported. In an effort to reduce the number of reads that align across alignment indices both BSSeeker2 and Bismark have strict default alignment parameters. In addition to being computationally demanding, this implementation can also reduce the number of valid alignments reported, as only the highest quality, unique alignments are output. BWA-Meth resolves this issue by performing alignment to a single bisulfite converted alignment index and processing reads on the fly; but, does not return the read level methylation calls or bisulfite conversion assessment provided by Bismark and BSSeeker2. Additionally, when performing bisulfite sequencing alignment the read conversion pattern is dependent on whether the sequenced DNA fragment is representative of the original DNA sequence or its PCR product. In a directional bisulfite sequencing library only DNA representative of the original DNA fragment is sequenced so the bisulfite conversion pattern is known. In an undirectional library, DNA representative of the original DNA fragment and its PCR product is sequenced so a cytosine to thymine or a guanine to adenine conversion is possible. BS-Seeker2 and Bismark handle undirecitonal libraries by converting input reads using both conversion patterns. This approach doubles the number of reads that must be aligned and generates input reads that will not be represented in the alignment index. BWA-Meth does not support alignment of undirectional libraries.

2. In my experience, installing pysam is more difficult than installing bwa-mem. So the statement "wrapping external read alignment tools introduces added complexity" is incorrect for example as it relates to bwa-meth. I expect the same is true for bismarck/bowtie(2). In addition to pysam, this tool seems to rely on samtools for methylation calling. That said, I was able to easily install this tool with pip.

Response:
We removed the statement about the added complexity of wrapping an alignment tool in the manuscript. Additionally, we have implemented Anaconda build recipes and added Anaconda installation instructions to the BSBolt documentation to provide a managed installation option.

3. This note:
"A read, or read pair, with a low proportion observed cytosines compared to guanine will be preferentially aligned
with a cytosine to thymine conversion pattern and vice versa. If it is unclear what conversion pattern should be used, both conversion patterns are aligned and the conversion pattern with the highest total alignment score is output." indicates the most important algorithmic improvement in BSBolt. A sentence indicating this strategy in the abstract would motivate the tool early on. Also please include additional detail on the exact value f "low proportion"

Response:
We added text to the manuscript to explicitly state what proportion level is set to by default and what was used to the comparison. Additionally, we highlighted the use of the read assessment in the abstract and several additional places throughout the paper.
A read, or read pair, with a low proportion of observed cytosines compared to guanine (0.1 by default) will be preferentially aligned with a cytosine to thymine conversion pattern and vice versa.

4. What is the motivation for this: " Each alignment and methylation
calling workflow was given a maximum runtime of 24 hours. If an alignment was incomplete at the end of 24 hours, duplicate read marking and methylation calling was performed on the reads aligned during the 24 hour limit. " ?
It would be clearer to let each tool complete in whatever time it takes and then report the time along with the full results.

Response:
We provided each tool with a maximum run time of 288 hours and updated the text accordingly (below). All incomplete Bismark alignments completed in this time, but several BSSeeker2 alignments were unfinished after the 288 hour limit. We acknowledge the unfinished alignments are a limitation of the manuscript, but it wasn’t feasible for us to extend the time limit beyond 288 hours.

5. Please add Table Legends
Response:

We added a table legend to table 1. Table 2 is represented as a figure in the revision and has captioned accordingly.

6. "The first 10kb of chr1 was duplicated and added as an additional contig."
This is all 'N' bases. What's the purpose of this?
Response:

The phrasing of the original sentence was incorrect. We duplicated the first 10kb of the simulated chr1. We clarified the text to highlight this.
The first 10kb of the simulated chr1 was duplicated and added as an additional contig.

7. nuttylogic.github.com/BSBoltManuscript is not available so I am not able to see the code to reproduce this analysis.
Likewise: https://bsbolt.readthedocs.io/ does not load (this might be an ephemeral clouflare issue).
I think this is the code used:
https://github.com/NuttyLogic/BSBoltManuscript/blob/master/AlignCompWGBS.py
In which case, if sam->bam conversion is used, it would be more fair to allow samtools view to use --threads if that is a bottleneck.

Response:

We evaluated if the samtools sam to bam conversion was a bottleneck for BISCUIT and BWA-METH. We also included BSBolt in the comparison as the conversion pipeline is built with htslib. There was performance gain when additional conversion threads were added. The addition of threads past 2 threads resulted in minimal performance gain so we set the conversion threads at 2. We also exposed the number of conversion threads as an option in BSBolt to prevent any bottlenecks for users. We now state this in the manuscript text and added a supplemental figure / text on this (text below).

Samtools and BSBolt were provided with two compression threads to minimize any alignment bottlenecks (supplemental figure 1).

8. In Table 2, please indicate that bwa-meth does not support undirectional and therefore the tool is not being used as intended.

Response:

We switched Table 2 with a figure in the revision and noted the BWA-Meth results accordingly.

Signed,
Brent Pedersen

Reviewer #2:
The authors present BSBolt, an analysis platform for processing bisulfite sequencing data. BSBolt introduces a new alignment file structure that allows rapid methylation calling. Benchmarking was performed using already existing tools, such as Bismark, BSSeeker2, BWA-Meth and BISCUIT. The BSBolt offers a very nice performance both in speed and accuracy.

Generally, the paper is well written, the results are clearly communicated. The BSBolt software is available with detailed documentation and a relatively easy installation. I needed to separately run 'make' for softwares in the External folder, maybe it worth mentioning in the documentation.
I have a few questions and suggestions:

Response:

We updated the github readme and documentation with more detailed installation instructions.

1. In the simulation, why did the authors use 0.05 as a mutation rate? If I interpret it correctly it is quite high, much higher than the general mutation rate for human. It might affect the performance of some tools, such as Bismark.

Response:

The mutation rate was set to 0.005 for the simulations, lower than 0.05, but it is certainly high compared to the expected human mutation rate and we expect around 0.5 genetic variants per 100bp. Directional reads simulated with a mutation rate of 0.005 and sequencing error rate of 0.005 were aligned accurately by both Bismark and BSSeeker2 (> 99.9%) with high mappability of 94.4% and 98.3% on average respectively (Table S1). Both Bismark and BSSeeker2 performed well at this baseline mutation rate. When the simulated error rates were increased to 0.02 BSSeeker2 and Bismark exhibited low mappability (Figure 2B) but the returned alignments were accurate (Table S1).

2. I was quite surprised by the low performance of Bismark. According to our experience, although slow and resource intensive, Bismark is quite accurate. In the simulation experiment the high mutation rate might explain this low performance, but it is the same with real data. Using similar computational setting, I don't recall Bismark taking us this long even with a somewhat bigger dataset. Did the authors check if the settings are adequate? The memory need increases quickly with the number of cores, can it be that it is limited by the amount of available memory? Using less cores might improve it. Are the accuracy results similar to those in the original publication about the dataset that was published (DOI:10.21203/rs.3.rs-33940/v1)? They also used Bismark there and compared it to Illumina array.

Response:
Bismark exhibited better performance when aligning the real data compared with the simulated read data. With the simulated directional, 100bp, paired-end reads Bismark performed the alignment in approximately 38 minutes (~35,500 reads / minute) compared with 3.239 minutes for BSBolt (~38X slower, table S2). With the real data Bismark aligned the libraries in 71.1 hours on average (~29,000 read pairs / minute) compared with BSBolt in 3.61 hours on average (~20X slower, table S3). In terms of accuracy, Bismarck, BSBolt, BISCUIT, and BWA-Meth all exhibited accuracy in line with previously reported results and manuscript text was updated to reflect this (text below). Additionally, outside of

BSSeeker2, all alignment tools showed low MAE between the sequencing methylation values and the array methylation values (Figure 3C).
The called methylation values were highly correlated with the sites called on the EPIC array across all alignment tools (Pearson's r=.92-98, supplemental table 2), as previously reported (Shu et al., 2020)

3. It would be interesting to see how BSBolt scales. What are the memory needs with 12 cores? Does it scale linearly? How fast it can be in a HPC environment with much more resources? It would be interesting to see a table or figure about it.

Response:

We added supplemental Figure 2 to show run time and memory consumption based on the number of alignment threads for single / paired end and directional / undirectional libraries 150bp libraries. Memory consumption increases linearly with the number of alignment threads. Run time decreases with added alignment threads, but the absolute run time is minimally changed by more threads after 8.

4. Despite it clearly shows good results, I think a more detailed rationale behind BSBolt would be nice, since BISCUIT offers very similar functionality with a slighter worse performance.

Response:

We expanded on the motivation to develop BSBolt in the manuscript text below. In summary, we had several goals with BSBolt. We sought to improve performance, eliminate the alignment constraints enforced by BSSeeker / BSSeeker2, preserve the read level methylation calling and read bisulfite conversion checks, and finally we wanted to take a minimal performance hit for alignment of undirectional libraries.
The three base alignment strategy as implemented by BSSeeker2 and Bismark has several limitations. Both tools carry out multiple intermediary alignments to separate alignment indices representing different reference conversion patterns and then integrate intermediate alignments together into a consensus alignment file. Reads with multiple alignments within an intermediate alignment file or across multiple intermediate alignment files are discarded; only reads that align uniquely within a single intermediate alignment are reported. In an effort to reduce the number of reads that align across alignment indices both BSSeeker2 and Bismark have strict default alignment parameters. In addition to being computationally demanding, this implementation can also reduce the number of valid alignments reported, as only the highest quality, unique alignments are output. BWA-Meth resolves this issue by performing alignment to a single bisulfite converted alignment index and processing reads on the fly; but, does not return the read level methylation calls or bisulfite conversion assessment provided by Bismark and BSSeeker2. Additionally, when performing bisulfite sequencing alignment the read conversion pattern is dependent on whether the sequenced DNA fragment is representative of the original DNA sequence or its PCR product. In a directional bisulfite sequencing library only DNA representative of the original DNA fragment is sequenced so the bisulfite conversion pattern is known. In an undirectional library, DNA representative of the original DNA fragment and its PCR product is sequenced so a cytosine to thymine or a guanine to adenine conversion is possible. BS-Seeker2 and Bismark handle undirecitonal libraries by converting input reads using both conversion patterns. This approach doubles the number of reads that must be aligned and generates input reads that will not be represented in the alignment index. BWA-Meth does not support alignment of undirectional libraries.

I also have some minor comments/recommendations:
I think table 2 would look better in a series of small figures, it would be quicker to go through the results.In the supplementary table 1, the "Aligned reads/min" should be "Million aligned reads/min".
Although python installation is easy, maybe it would worth making it available in conda or as a docker container for smoother integration in different environments.

Response:

We removed Table 2 and added a figure summarizing the results in its place. The labels for supplementary table 1 have been fixed. Additionally, we added conda build recipes for macOS and linux 64 to ease installation.

Close
