# Supplementary material for: BiSulfite Bolt: A bisulfite sequencing analysis platform
Source: Gigascience. 2021 May 8;10(5):giab033. doi: 10.1093/gigascience/giab033 (PMC8106542; doi:10.1093/gigascience/giab033)
Supplement: giab033_Reviewer_1_Report_Original_Submission — Brent Pedersen -- 10/30/2020 Reviewed [file giab033_reviewer_1_report_original_submission.pdf]

Reviewer Report

Title: BiSulfite Bolt: A BiSulfite Sequencing Analysis Platform

Version: Original Submission **Date:** 10/30/2020

Reviewer name: Brent Pedersen

Reviewer Comments to Author:

The authors introduce BSBolt as a complete pipeline for processing bisulfite sequence data.
The main stated contribution of this tool over the authors' previous work of BSSeeker and BSSeeker2 is the direct modification of BWA-mem to align BS-Seq reads directly which results in increased accuracy.
This seems like a nice improvement over existing methods. Several clarifications/changes in the paper would be helpful.

1. Given that the authors have previously written BSSeeker and recently written BSSeeker2, more concise motivation of what short-coming BSBolt addresses in specifically those two tools would be helpful.
2. In my experience, installing pysam is more difficult than installing bwa-mem. So the statement "wrapping external read alignment tools introduces added complexity" is incorrect for example as it relates to bwa-meth. I expect the same is true for bismarck/bowtie(2). In addition to pysam, this tool seems to rely on samtools for methylation calling. That said, I was able to easily install this tool with pip.

3. This note:
"A read, or read pair, with a low proportion observed cytosines compared to guanine will be preferentially aligned
with a cytosine to thymine conversion pattern and vice versa. If it is unclear what conversion pattern should be used, both conversion patterns are aligned and the conversion pattern with the highest total alignment score is output."
indicates the most important algorithmic improvement in BSBolt. A sentence indicating this strategy in in the abstract would motivate the tool early on. Also please include additional detail on the exact value
of "low proportion"
4. What is the motivation for this: " Each alignment and methylation calling workflow was given a maximum runtime of 24 hours. If an alignment was incomplete at the end of
24 hours, duplicate read marking and methylation calling was performed on the reads aligned during the
24 hour limit. " ?
It would be clearer to let each tool complete in whatever time it takes and then report the time along with the full results.

5. Please add Table Legends
6. "The first 10kb of chr1 was duplicated and added as an additional contig."
  This is all 'N' bases. What's the purpose of this?
7. nuttylogic.github.com/BSBoltManuscript is not available so I am not able to see the code to reproduce this analysis.
  Likewise: https://bsbolt.readthedocs.io/ does not load (this might be an ephemeral clouflare issue).
  I think this is the code used:
  https://github.com/NuttyLogic/BSBoltManuscript/blob/master/AlignCompWGBS.py
  In which case, if sam->bam conversion is used, it would be more fair to allow samtools view to use --threads
  if that is a bottleneck.
8. In Table 2, please indicate that bwa-meth does not support undirectional and therefore the tool is not being used as intended.
Signed,
Brent Pedersen

Level of Interest

Please indicate how interesting you found the manuscript: Choose an item.

Quality of Written English

Please indicate the quality of language in the manuscript: Choose an item.

Declaration of Competing Interests

Please complete a declaration of competing interests, considering the following questions:

- Have you in the past five years received reimbursements, fees, funding, or salary from an organisation that may in any way gain or lose financially from the publication of this manuscript, either now or in the future?
- Do you hold any stocks or shares in an organisation that may in any way gain or lose financially from the publication of this manuscript, either now or in the future?
- Do you hold or are you currently applying for any patents relating to the content of the manuscript?
- Have you received reimbursements, fees, funding, or salary from an organization that holds or has applied for patents relating to the content of the manuscript?
- Do you have any other financial competing interests?
- Do you have any non-financial competing interests in relation to this paper?

If you can answer no to all of the above, write 'I declare that I have no competing interests' below. If your reply is yes to any, please give details below.

I declare that I have no competing interests

I agree to the open peer review policy of the journal. I understand that my name will be included on my report to the authors and, if the manuscript is accepted for publication, my named report including any attachments I upload will be posted on the website along with the authors' responses. I agree for my report to be made available under an Open Access Creative Commons CC-BY license (http://creativecommons.org/licenses/by/4.0/). I understand that any comments which I do not wish to be included in my named report can be included as confidential comments to the editors, which will not be published.

Choose an item.

To further support our reviewers, we have joined with Publons, where you can gain additional credit to further highlight your hard work (see: https://publons.com/journal/530/gigascience). On publication of this paper, your review will be automatically added to Publons, you can then choose whether or not to claim your Publons credit. I understand this statement.

Yes Choose an item.
