# Supplementary material for: BiSulfite Bolt: A bisulfite sequencing analysis platform
Source: Gigascience. 2021 May 8;10(5):giab033. doi: 10.1093/gigascience/giab033 (PMC8106542; doi:10.1093/gigascience/giab033)
Supplement: giab033_Reviewer_2_Report_Original_Submission — Reka Toth -- 11/12/2020 Reviewed [file giab033_reviewer_2_report_original_submission.pdf]

Reviewer Report

Title: BiSulfite Bolt: A BiSulfite Sequencing Analysis Platform

Version: Original Submission **Date:** 11/12/2020

Reviewer name: Reka Toth

Reviewer Comments to Author:

The authors present BSBolt, an analysis platform for processing bisulfite sequencing data. BSBolt introduces a new alignment file structure that allows rapid methylation calling. Benchmarking was performed using already existing tools, such as Bismark, BSSeeker2, BWA-Meth and BISCUIT. The BSBolt offers a very nice performance both in speed and accuracy.
Generally, the paper is well written, the results are clearly communicated. The BSBolt software is available with detailed documentation and a relatively easy installation. I needed to separately run 'make' for softwares in the External folder, maybe it worth mentioning in the documentation.
I have a few questions and suggestions:
1. In the simulation, why did the authors use 0.05 as a mutation rate? If I interpret it correctly it is quite high, much higher than the general mutation rate for human. It might affect the performance of some tools, such as Bismark.
2. I was quite surprised by the low performance of Bismark. According to our experience, although slow and resource intensive, Bismark is quite accurate. In the simulation experiment the high mutation rate might explain this low performance, but it is the same with real data. Using similar computational setting, I don't recall Bismark taking us this long even with a somewhat bigger dataset. Did the authors check if the settings are adequate? The memory need increases quickly with the number of cores, can it be that it is limited by the amount of available memory? Using less cores might improve it. Are the accuracy results similar to those in the original publication about the dataset that was published (DOI:10.21203/rs.3.rs-33940/v1)? They also used Bismark there and compared it to Illumina array.
3. It would be interesting to see how BSBolt scales. What are the memory needs with 12 cores? Does it scale linearly? How fast it can be in a HPC environment with much more resources? It would be interesting to see a table or figure about it.
4. Despite it clearly shows good results, I think a more detailed rationale behind BSBolt would be nice, since BISCUIT offers very similar functionality with a slighter worse performance.
I also have some minor comments/recommendations:
I think table 2 would look better in a series of small figures, it would be quicker to go through the results.
In the supplementary table 1, the "Aligned reads/min" should be "Million aligned reads/min".
Although python installation is easy, maybe it would worth making it available in conda or as a docker container for smoother integration in different environments.

Level of Interest

Please indicate how interesting you found the manuscript: Choose an item.

Quality of Written English

Please indicate the quality of language in the manuscript: Choose an item.

Declaration of Competing Interests

Please complete a declaration of competing interests, considering the following questions:

- Have you in the past five years received reimbursements, fees, funding, or salary from an organisation that may in any way gain or lose financially from the publication of this manuscript, either now or in the future?
- Do you hold any stocks or shares in an organisation that may in any way gain or lose financially from the publication of this manuscript, either now or in the future?
- Do you hold or are you currently applying for any patents relating to the content of the manuscript?
- Have you received reimbursements, fees, funding, or salary from an organization that holds or has applied for patents relating to the content of the manuscript?
- Do you have any other financial competing interests?
- Do you have any non-financial competing interests in relation to this paper?

If you can answer no to all of the above, write 'I declare that I have no competing interests' below. If your reply is yes to any, please give details below.

I declare that I have no competing interests

I agree to the open peer review policy of the journal. I understand that my name will be included on my report to the authors and, if the manuscript is accepted for publication, my named report including any attachments I upload will be posted on the website along with the authors' responses. I agree for my report to be made available under an Open Access Creative Commons CC-BY license (http://creativecommons.org/licenses/by/4.0/). I understand that any comments which I do not wish to be included in my named report can be included as confidential comments to the editors, which will not be published.

Choose an item.

To further support our reviewers, we have joined with Publons, where you can gain additional credit to further highlight your hard work (see: https://publons.com/journal/530/gigascience). On publication of this paper, your review will be automatically added to Publons, you can then choose whether or not to claim your Publons credit. I understand this statement.

Yes Choose an item.
